# Analysis of a Smartphone-Based Architecture with Multiple Mobility Sensors for Fall Detection with Supervised Learning

**DOI:** 10.3390/s18041155

**Published:** 2018-04-10

**Authors:** José Antonio Santoyo-Ramón, Eduardo Casilari, José Manuel Cano-García

**Affiliations:** Departamento de Tecnología Electrónica, Universidad de Málaga, ETSI Telecomunicación, 29071 Málaga, Spain; jasantoyo@uma.es (J.A.S.-R.); jcgarcia@uma.es (J.M.C.-G.)

**Keywords:** fall detection system, inertial sensors, smartphones, accelerometers, machine learning algorithms, supervised learning, ANOVA analysis

## Abstract

This paper describes a wearable Fall Detection System (FDS) based on a body-area network consisting of four nodes provided with inertial sensors and Bluetooth wireless interfaces. The signals captured by the nodes are sent to a smartphone which simultaneously acts as another sensing point. In contrast to many FDSs proposed by the literature (which only consider a single sensor), the multisensory nature of the prototype is utilized to investigate the impact of the number and the positions of the sensors on the effectiveness of the production of the fall detection decision. In particular, the study assesses the capability of four popular machine learning algorithms to discriminate the dynamics of the Activities of Daily Living (ADLs) and falls generated by a set of experimental subjects, when the combined use of the sensors located on different parts of the body is considered. Prior to this, the election of the statistics that optimize the characterization of the acceleration signals and the efficacy of the FDS is also investigated. As another important methodological novelty in this field, the statistical significance of all the results (an aspect which is usually neglected by other works) is validated by an analysis of variance (ANOVA).

## 1. Introduction

According to some forecasts [1], it is expected that between 2015 and 2050 the over-60 years of age world population will grow from 900 to 2000 million. This dramatic demographic change will undoubtedly give rise to a series of challenges in the health systems that must be faced in order to sustain and increase the quality of life of the citizens. The present study focuses on one of the biggest public health problems faced by the world society: falls.

A fall can result in minor consequences such as bruises, lacerations or abrasions, but it can also lead to more serious and dangerous situations such as bone fractures or psychological disorders (such as the so-called Fear of Falling or FoF syndrome). The World Health Organization has reported that falls are the second worldwide cause of mortality provoked by accidental or unintentional injuries. 37.3 million falls requiring medical attention occur annually, while it has been estimated that around 646,000 people die every year due to falls [2] A remarkable fact is that people over 65 years old experience the greatest number of falls. Approximately 28–35% of people over 65 suffer a fall each year, whereas this percentage climbs with age, reaching 32–42% for people over 70 [3]. Risk groups are not limited to the older people. Other population groups are exposed to endure severe falls during their work or leisure time (cyclists, mountaineers, firemen, antenna installers, cable layers, etc.).

The probability of survival after a serious fall is strongly related to the rapidness of the medical assistance. As mentioned in [4], 50% of people affected by a bad fall who remain without assistance for more than an hour die before the six months following the accident. Consequently, the research on reliable and cost-effective systems for the automatic detection of falls has gained attention during the last decade.

## 2. State of the Art on Wearable Fall Detection Systems and Multisensory Architectures

Fall Detection Systems (FDS) are intended to permanently monitor the mobility of a target user or patient in order to automatically alert his/her relatives or the medical staff whenever a fall occurrence is identified. The goal of a FDS is to maximize the possibility of detecting actual falls while minimizing the number of false alarms, i.e., the conventional movements or Activities of Daily Living (ADLs) that are mistaken as falls.

FDSs can be generically classified into two large groups. On the one hand, in context-aware systems, the detection decision is based on the signals captured by a network of environmental sensors (microphones, cameras, vibration sensors, etc.) located in a specific area around the user to be monitored. These FDSs have clear drawbacks provoked by their high installation and maintenance costs, their limitation in terms of the area where the user can be monitored or their high probability of spurious interference caused by external factors such as changes in the light, noises, presence of another individual, displacement of furniture, domestic pets or other falling objects, etc. In addition, under a context-aware tracking solution, the user can feel their privacy invaded because of the continuous use of audiovisual equipment.

In contrast, wearable systems use sensors that are integrated into the patient’s clothing. Hence, these sensors only track magnitudes unambiguously linked to the patient mobility, such as the acceleration or angular velocity of the body. Wearable systems offer some clear advantages over the context-aware solutions since the restrictions about the monitoring area are eliminated, as long as the sensors always accompany the patient. Besides, they are also less intrusive, more economical and less vulnerable to the effects of external factors. As disadvantages, wearable FDSs can adversely affect the patients’ comfort during their daily routines (especially if they are too bulky). They can also be useless if the user forgets to wear it or to recharge the corresponding battery or if the FDS is unintentionally misplaced or not fit properly on the patient’s body. In addition, in certain environments such as toilets or bathrooms or during certain activities (such as showers) the monitoring activity may be inviable.

Due to the fact that most present smartphones integrate the inertial sensors that are required by a wearable FDS (mainly an accelerometer but also a gyroscope and a magnetometer), smartphone technology has been proposed as a basis to deploy cost-effective FDSs [5,6]. The use of smartphones can benefit from the plummeting costs, increasing hardware capabilities and the widespread popularity of these personal devices. Although the access to a mobile phone is lower for older age groups, smartphone usage among people over 60 years old in the United States was about 81% in 2005, while 60% of the elderly above 60 declare to own a smartphone [7]. Moreover, these percentages tend to increase. As predicted in [8], almost the entire population over 60 years old in Western countries is expected to use cell phones in the next 10 years.

As a result of the easiness of developing a FDS on a smartphone, most works of the recent related literature on wearable FDS have focused on the study of ‘smartphone-only’ based detectors, i.e., architectures that employ the smartphone as the unique element in the system, simultaneously acting as a sensing unit, data processor node and communication gateway [9]. As smartphones are natively provided with cellular (3G/4G) communications, the patient can be tracked almost ubiquitously (at least in urban scenarios) and fall detection alarms can be seamlessly integrated with emergency response systems by means of a SMS, an automatic phone call to a predefined set of phone numbers, a notification through a remote server on the Internet, etc. The use of smartphones as mobility monitoring units have been even proposed to characterize the gait speed as a predictor of post-operative morbidity and mortality in elderly cardiovascular disease [10]. 

However, it has been have shown [11] that the position of the sensor that monitors the movements of the subject is crucial for the effectiveness of the FDS. In this regard, recommended locations such as the chest or waist are not comfortable positions to place a smartphone (which is conventionally transported in a hand-bag or in a loose pocket, where the phone may exhibit a certain freedom of movements that can affect the representativeness of the mobility measurements provided by its sensors). Furthermore, the range of the sensors integrated in the smartphone were not originally conceived to quantify the intensity of the movements that a fall can produce. Thus, the use of smaller and specific sensors that can be easily incorporated into the patient’s clothing seems advisable for the sake of ergonomics. Nowadays there is a great variety of low-cost sensing motes that can be used for this purpose. These devices embed inertial sensors accelerometers as well as wireless communication interfaces (Bluetooth, 802.15.4 ...) that can be utilized to send the monitored signals to devices with a greater processing capacity such as a smartphone.

Multisensory Body Area Networks (BANs) have been proposed in works such as [12,13,14] to recognize and differentiate diverse activities of daily life. These networks are composed of a set of sensing nodes located on different parts of the body of the subjects under test. Nonetheless, in these works the network was never employed to identify falls and in most of them the importance of the position of the sensors to identify the activities was not specifically addressed as a research issue.

The work by Hyunh et al. in [15] (whose results are commented by the same authors in [16]) investigated the optimal position of a fall detection sensor, concluding that the sensor should be attached to the center of the chest.

An architecture with multiple sensors for fall detection has been recently investigated by Turan in the interesting work presented in [17]. This author analyzed the performance of a FDS built on a BAN consisting of six wireless motes placed at six positions (head, chest, waist, wrist, thigh and ankle). After recording a dataset of falls and movements of daily life executed by a group of experimental subjects, the effectiveness of different detection algorithms to discriminate falls is assessed taking into account the positions of the motes that are considered to produce the detection decision. In the deployed testbed, the network did not employ any smartphone (the signals are sent via ZigBee to a computer) while the ADLs emulated by the volunteers did not include activities (such as hopping or climbing stairs) that can be easily mistaken as falls. The statistical significance of the results was not evaluated either. In fact, except for a very reduced set of works (such as [18], in which a *p*-value analysis is carried out to examine if the election of the samples influences the efficacy of the detection method), the literature on FDSs disregards the analysis of the statistical significance of the metrics that are obtained to compare the performance of the detection algorithms.

The present study will extend a previous work [11] to systematically evaluate the application of different learning machine strategies when they are applied to the traces captured by a hybrid multisensory FDS architecture (consisting of a set of sensing motes and a smartphone). The study tries to optimize the election of the acceleration statistics that characterize the body mobility as well as to identify the sensor placement combinations that produce the best detection performance. In all the cases, the validity of the conclusions will be contrasted through an analysis of variance (ANOVA) of the results.

## 3. Description of the Experimental Testbed

**Figure 1:**
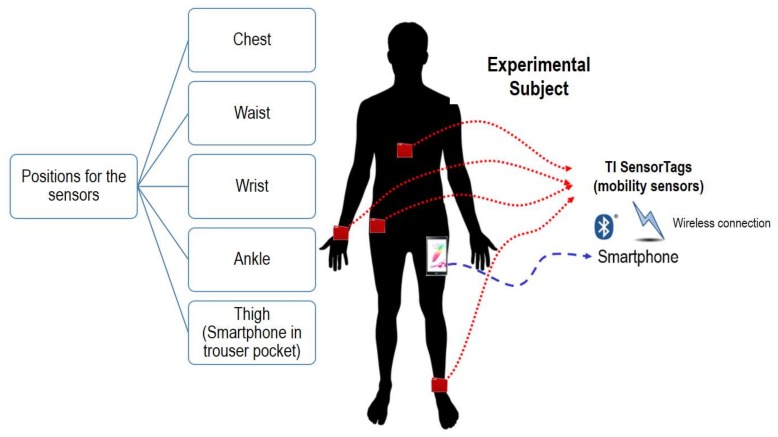
Basic architecture of the testbed.

In order to characterize the movements of a set of experimental subjects, we developed a monitoring system, sketched in Figure 1, based on a smartphone which is wirelessly connected to a set of four sensing nodes or ‘motes’, which are located on different parts of the body of the subject (chest, waist, wrist and ankle). The motes measure the acceleration (expressed in *g* –or g-force-), angular velocity (in °/s) and flux density of the magnetic field (in µT).

The sensing nodes were deployed on CC2650 SimpleLink™ Bluetooth low energy/Multi-standard SensorTag modules [19], manufactured by Texas Instruments. Every SensorTag incorporates a CC2650 ARM microcontroller and a set of MEMS sensors, including an InveSense MPU-9250 Inertial Measurement Unit (IMU) which integrates three triaxial sensors: an accelerometer, a gyroscope and a magnetometer. The SensorTag modules, which are powered through a CR2032-type battery, support different wireless communications, which avoids wiring and provides the user with a complete freedom of movement. In particular, the sensing motes embed a multi-standard 2.4 GHz ultra-low power wireless MCU compatible with ZigBee, 6LowPAN or Bluetooth Low Energy (BLE) communications. In our prototype, benefiting from the fact that most current smartphones support BLE, a smartphone was selected as the central node of a BLE piconet. The piconet follows a typical star topology. Thus, the smartphone, which plays the role of the piconet master, receives all the packets containing the measurements captured by four SensorTags, which act as slaves in the BLE piconet. 

The best election of the sensor sampling rate is a still open issue in the area of fall detection system (see the works by Medrano in [20] and Fudickar in [21] for a further analysis of the importance of the sampling rate in the efficacy of a FDS). As a matter of fact, sampling frequencies ranging from 5 Hz to 256 Hz have been used by the related literature to generate benchmarking datasets to compare fall detection algorithms. Aiming at avoiding saturation problems in the Bluetooth network, the sampling rate of the sensors was set to 20 Hz. For that purpose, the original firmware of the SensorTags was modified, so that the 9 values periodically collected by the three IMU triaxial sensors could be transmitted every 50 ms through the BLE connection. In addition, as the smartphone in turn also integrates an IMU, we used the mobile phone, which was located in a trouser pocket for all the experiments, as a fifth sensor to describe the mobility registered on the subject’s thigh. The employed sampling frequency of the smartphone measurement was 200 Hz.

Besides, a specific Android application (app) was programmed to receive and save the information captured by all the sensors, so that a repository with the generated mobility traces could be progressively generated. The app is in charge of storing the measurements transmitted from the four SensorTags as well as those captured by the smartphone in a CSV (Comma Separated Value)-format log file. Every sample received from any of the mobility sensors and any source (the four SensorTags and the smartphone) is recorded in an independent line of the log file, together with a timestamp generated by the app. No further synchronization between the sensing nodes is needed as long as a default value of 30 ms is selected by Android OS for the polling BLE Connection Interval (the time between two consecutive transmissions from the same slave to the Bluetooth master). So, the communication delay that may be introduced by BLE technology can be considered negligible when compared to the observation window that is required to detect a fall event.

For each experiment the program creates a new log file that stores the signals obtained from the subject during the execution of a certain movement (ADL or fall) for a fixed period of time (15 s). The log file also includes the personal characteristics of the experimental subject (weight, age, gender, height) as well as the typology of the experiment performed movement. In the log files, whenever a sample is received from a certain sensor, the app adds a timestamp and the MAC address of the mote (so that the sensor that has sent the information can be identified). Finally, in every log file, the app includes the employed sampling rate and additional information of the sensing device (manufacturer, range, etc.).

The final resulting dataset (called UMAFall) containing all the log files has been made publicly available in Internet [22] (together with some video clips that illustrate the activity of the subjects) as a benchmarking tool for the research on fall detection systems.

With regard to energy consumption, the batteries of the sensors allowed generating the dataset without being replaced. Similarly, the battery of the smartphone supported experimental one-day sessions of continuous use without having to be recharged. 

Battery-life is obviously a key issue for the feasibility of any wearable monitoring system. However, the detailed study of the consumption of the implemented architecture is out of the analysis of this work (which focuses on evaluating the performance of the detection algorithms). Refer to [23,24,25] for a further study of the challenges caused by mobile sensing to battery-life in wearables and smart personal devices.

As it respects to the employed methodology to validate the detection strategies, due to the obvious difficulties of utilizing measurements from real-world falls, we followed the procedure that is commonly observed by the related literature. Thus, a group of 19 experimental subjects, whose basic characteristics are presented in Table 1, systematically performed a series of predefined movements, including 11 types of Activities of Daily Life (ADL) and 3 types of emulated falls, which were executed on a mattress to avoid injuries. Table 2 in Section 5 includes a description of each type of movement as well as the number of samples collected from the subjects. For the selection of the typology of ADLs and falls in the testbed we considered the following criteria:

- To include the falls and ADLs that are typically considered in similar datasets in the literature (see [26] for a detailed analysis of these existing datasets). This typical ADLs encompass from basic operation (walking, sitting down) to daily activities which may cause very specific patterns of the acceleration measurements (applauding, going upstairs or downstairs).
- To incorporate some ‘sporting activities’ (hopping, running), normally neglected by all these datasets, which may cause strong acceleration peaks that must be discriminated from those originated during a fall event.

The divergence in the mobility patterns of actual falls suffered by older people and falls mimicked by young and healthy adults on a cushioning surface is still under discussion (see, for example, the studies in [27,28,29]) but that issue is beyond the scope of this paper. In any case, as it can be noticed from Table 1, two experimental subjects older than 65 participated in our testbed although they did not perform falling movements for safety reasons.

**Figure 2:**
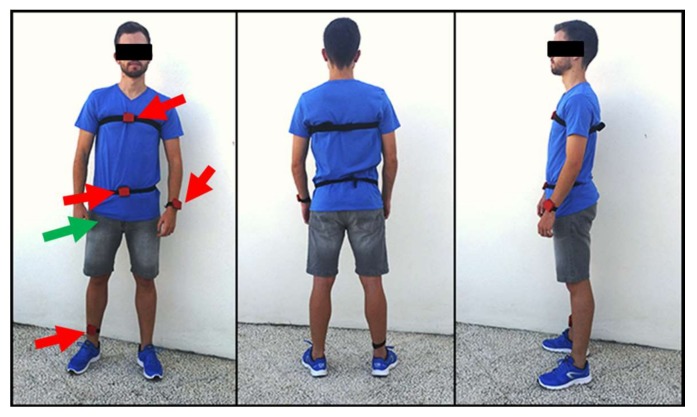
Example of the distribution of the sensors worn by an experimental subject. The green arrow indicates the position of the smartphone. Red arrows correspond to the SensorTag modules, which are attached to the user’s body by means of elastic bands.

**Figure 3:**
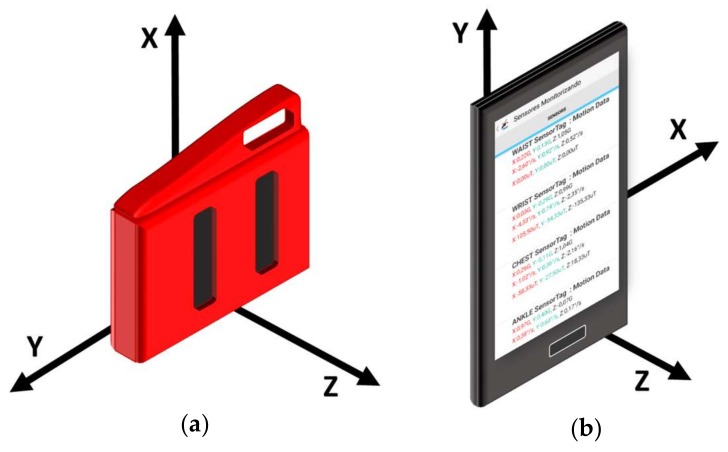
Representation of the spatial reference system of the employed sensing devices (devices are firmly attached to the subjects’ body to guarantee that the reference system does not change during the experiments). (**a**) SensorTag (**b**) Smartphone.

In all the trials, the starting position of the subject performing the movement (ADL or fall) was standing with hands on hips. All the experiments took place in a home environment. As it can be contemplated in Figure 2, the motes were tightly attached to the subject’s body by means of elastic bands. For all the tests, the accelerometer axes of the SensorTag modules and the Smartphone were oriented and aligned as depicted in Figure 3. Thus, the components of the measurements of the tri-axial sensors are always associated to the same actual direction of the body position.

The log files obtained (one for each experiment) were formatted and stored in the internal memory of the smartphone. After the experiments were finished, the data were extracted and moved to a PC where they are processed in an offline way with Matlab scripts that implement the different machine learning algorithms to be tested.

## 4. Machine Learning Algorithms and Selection of the Input Features

**Figure 4:**
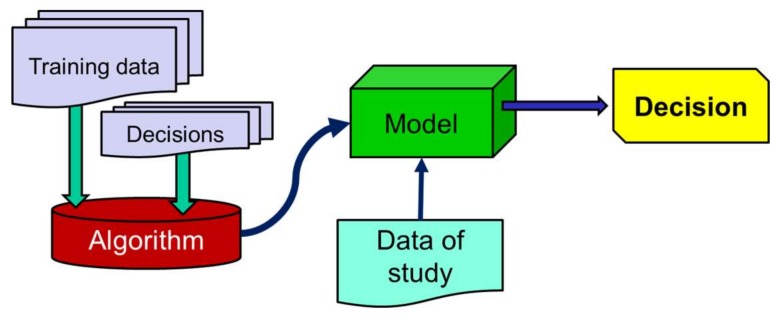
Typical Flow chart of a supervised learning classification algorithm.

The data collected through the developed system are processed and subsequently utilized to study the behavior of four supervised learning classification algorithms. Such algorithms (whose operational procedure is sketched in Figure 4) need to be trained before being applied to the test data. This training is achieved by providing the algorithm with a series of features (statistics that characterize the movements and which are computed from the mobility traces) that are extracted from a set of training samples as well as with the decisions that the detector should make (ADL or fall) for every sample. From the training phase, the algorithm builds a mathematical model that is later employed with the test data to decide the data ‘class’, that is to say, to discriminate falls from ADLs [30].

### 4.1. Feature Extraction: Selection of the Input Statistics of the Machine Learning Algorithms

In the development of any machine learning algorithm, a proper selection of the input characteristics is a key aspect.

As in most works in the literature, the statistical characterization of the movements will be based on the measurements ($A_{X_{i}}$, $A_{Y_{i}}$, $A_{Z_{i}}$) of the acceleration captured by the tri-axial accelerometers embedded in the five sensing points: chest, wrist, waist, ankle and thigh (pocket). Future studies should contemplate the information provided by the gyroscope and the magnetometer.

The statistics to be studied as the input of the supervised algorithms are derived from the samples of 15 s generated through the experimental testbed. Falls are typically associated to a brusque decay (which tends to 0 g) of the acceleration components (during an initial free-fall period) followed by one or several acceleration peaks caused by the impact (or impact) against the floor [31]. Thus, we focus our analysis of the signal on the period of the signal where the difference between the ‘peaks’ and the valleys’ in the acceleration is maximized. In particular, to analyze the acceleration measurements in every axis (*x*, *y* or *z*), we utilize a sliding window of duration *t_W_* = 0.5 s or *N_W_* samples, where:

(1) $$N_{W}=t_{W}\cdot f_{s}$$

being *f_s_* the sampling rate of the sensors (20 Hz for the SensorTags and 200 Hz for the smartphone). This value of 0.5 s is coherent with other related studies on context aware and wearable systems for fall detection, which also employ a sliding window to detect the falls: 0.4 s [32], 0.6 s [33], 0.75 s [34], 1 s in [35,36] or 2 s in [17,37,38]. In [39] authors claim that an observation interval of 0.5 is the best compromise between effectiveness, complexity and low power consumption to track the acceleration measurements in a fall detection system.

In order to locate the interval where the acceleration components suffer the highest variation, we compute for each window the module of the maximum variation of the acceleration components in the three axes. For the *j*-th window, this parameter ($A_{w_{diff}}\left(j\right)$) is calculated as:

(2) $$A_{w_{diff}}\left(j\right)=\sqrt{{\left(A_{X_{\max_{j}}}-A_{X}{}_{\min_{j}}\right)}^{2}+{\left(A_{Y_{\max_{j}}}-A_{Y}{}_{\min_{j}}\right)}^{2}+{\left(A_{Z_{\max_{j}}}-A_{Z}{}_{\min_{j}}\right)}^{2}}$$

where $A_{X_{\max_{j}}}$, $A_{Y_{\max_{j}}}$ and $A_{Z_{\max_{j}}}$ indicate the maximum values of the acceleration components in the *x*, *y* and *z-* axes during the *j*-th sliding window (e.g., $A_{X_{\max_{j}}}=\max\left(A_{X_{i}}\right)\;\forall i\in \left[j,j+N_{W}-1\right]$).

Thus, the analysis interval will be defined as the subset of consecutive samples $\left[k_{o},k_{o}+N_{W}-1\right]$ where the maximum value of $A_{w_{diff}}\left(j\right)$ is found to be:

(3) $$A_{w_{diff\left(max\right)}}=A_{w_{diff}}\left(k_{o}\right)=\max\left(A_{w_{diff}}\left(j\right)\right)\;\forall j\in \left[1,N-j\right])$$

where *k_o_* is the first sample of the analysis interval while *N* indicates the total number of samples of the trace (for each axis).

The rest of the input features for the detection algorithms are computed just taking into account the values of the acceleration components during this analysis interval. We consider as candidate features to feed the machine learning algorithms the following statistics:

- Mean Signal Magnitude Vector ($\mu_{SMV}$), which describes the mean motion or agitation level of the body throughout the movement. This variable is computable as the mean module of the acceleration vector during the analysis interval:
  (4) $$\mu_{SMV}=\frac{1}{N_{W}}\cdot \;\overset{k_{o}+N_{W}-1}{\underset{i=k_{o}}{\sum }}SMV_{i}$$
  where *SMV_i_* represents the Signal Magnitude Vector for the *i*-th measurement of the accelerometer:
  (5) $$SMV_{i}=\sqrt{A_{X_{i}}{}^{2}+A_{Y_{i}}{}^{2}+A_{Z_{i}}{}^{2}\;}$$
- The standard deviation of the Signal Magnitude Vector ($\sigma_{SMV}$), which informs about the variability of the acceleration
  (6) $$\sigma_{SMV}=\sqrt{\frac{1}{N_{W}}\cdot \;\overset{k_{o}+N_{W}-1}{\underset{i=k_{o}}{\sum }}{\left(SMV_{i}-\mu_{SMV}\right)}^{2}}$$
  This parameter may be clearly affected by the presence of ‘valleys’ and ‘peaks’ in the evolution of the acceleration.
- The sudden fluctuation of the mobility during a fall can be also described by the mean absolute difference ($\mu_{SMV_{diff}}$) between consecutive samples of the acceleration module [40]:
  (7) $$\mu_{SMV_{diff}}=\frac{1}{N_{W}}\cdot \;\overset{k_{o}+N_{W}-1}{\underset{i=k_{o}}{\sum }}\left|SMV_{i+1}-SMV_{i}\right|$$
- As a fall occurrence usually implies a change in the orientation of the body, we also consider the mean rotation angle ($\mu_{\theta }$), computable as [40]:
  (8) $$\mu_{\theta }=\frac{1}{N_{W}}\cdot \;\overset{k_{o}+N_{W}-1}{\underset{i=k_{o}}{\sum }}{\left(\cos^{-1}\left[\frac{A_{X}{}_{i}\cdot A_{X}{}_{i+1}+A_{Y}{}_{i}\cdot A_{Y}{}_{i+1}+A_{Z}{}_{i}\cdot A_{Z}{}_{i+1}\;}{SMV_{i}\cdot SMV_{i+1}\;}\right]\right)}^{}$$
- While the subject remains in an upright position, the effect of the gravity strongly determines the value of the acceleration component in the direction which is perpendicular to the floor plane. As a consequence, the inclination of the body caused by the falls normally provokes a remarkable modification of the acceleration components that defines the plane parallel to the floor when the subject is standing. Thus, to characterize this phenomenon, we utilize as a new feature the mean module ($\mu_{Ap}$) of these acceleration components ($A_{Y}$ and $A_{Z}$ in the case of the SensorTag motes, and $A_{X}$ and $A_{Z}$ for the smartphone, as it can be appreciated from the resting upright position depicted in Figure 2).
  (9) $$\mu_{Ap}=\frac{1}{N_{W}}\cdot \;\overset{k_{o}+N_{W}-1}{\underset{i=k_{o}}{\sum }}\sqrt{A_{Y_{i}}{}^{2}+A_{Z_{i}}{}^{2}\;}\mathrm{for}\;\mathrm{the}\;\mathrm{SensorTags}$$
  (10) $$\mu_{Ap}=\frac{1}{N_{W}}\cdot \;\overset{k_{o}+N_{W}-1}{\underset{i=k_{o}}{\sum }}\sqrt{A_{X_{i}}{}^{2}+A_{Z_{i}}{}^{2}\;}\mathrm{for}\;\mathrm{the}\;\mathrm{smartphone}$$

### 4.2. Employed Supervised Learning Classification Algorithm

As the detection techniques, we compare four supervised learning classification algorithms that are commonly employed by the related literature (refer to [5,8,41,42,43,44,45] for a state-of-the-art on the detection techniques used in FDS): Support Vector Machine, *k*-Nearest Neighbors, Naives Bayes and Decision Tree, which are briefly presented in the following sub-sections (see [30,46] to gain a much deeper insight into this set of techniques).

#### 4.2.1. Support Vector Machine (SVM)

Support Vector Machine (SVM) is perhaps the most popular learning algorithm employed in the fall detection system literature [31,47,48,49,50,51]. According to the SVM algorithm, the input space defined trough the features of the different training samples is converted into a multi-dimensional space by means of a non-linear mapping. 

**Figure 5:**
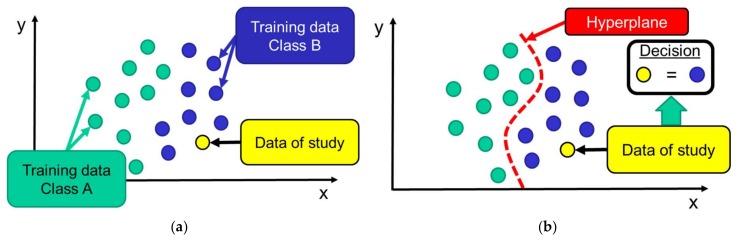
Example of the performance of the SVM algorithm for a two-dimensional space (samples characterized by two input features): (**a**) distribution of the training data on the two-dimensional space (**b**) creation of the hyperplane and classification decision for a certain test data.

Thus, as reflected in Figure 5, from the training dataset the algorithm is capable of building a ‘maximum margin hyperplane’ that acts as a decision boundary to categorize and discriminate the samples. The hyperplane, which separates two multi-dimensional regions, is defined so that the distances to the nearest points (from both sides) are maximized [30]. Once that the system is trained, the classification of the samples is directly based on the region where the sample is included. 

#### 4.2.2. *k*-Nearest Neighbors (*k*-NN)

**Figure 6:**
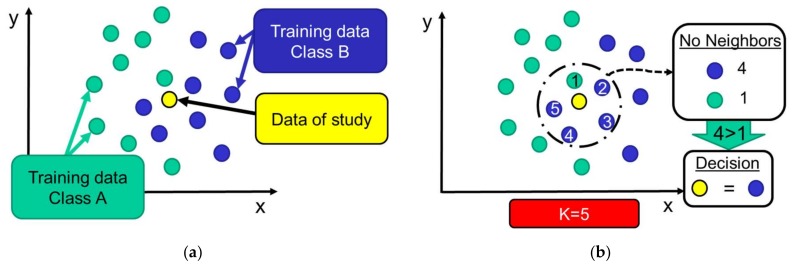
Operation of *k*-NN classifier: (**a**) the data under study is located among the training patterns aiming at finding the *k* nearest neighbor (in the example *k* = 5); (**b**) after detecting the *k* nearest samples, the new sample is classified considering the ‘majority vote’ (most common class) of its neighbors.

This instance-based classifier has been utilized as a decision algorithm in works such as [17,50,51,52,53,54]. The typical operation of *k*-NN is represented in Figure 6, utilizes the training dataset in a very simple way: whenever a new activity has to be classified, *k*-NN searches for the *k* already classified samples that are closest to this new uncategorized data. After this, the classification decision is based on the most popular class that is found among these neighbors. Although other techniques (such as Manhattan and city-block distances) have been suggested [42], we compute the Euclidean distance between the numerical features to estimate the distance between the training samples and the sample to be classified.

#### 4.2.3. Naive Bayes

This probabilistic classifier (used for FDS in papers such as [50,55]) calculates the probabilities of a particular sample to belong to the existing classes as a function of its associated multi-variate features. By applying the Bayes Theorem, these probabilities are estimated from the prior probability of the classes and the features, as well as the so-called *likelihood* (conditional probability of the features given a class). For this purpose, the statistical characterization of these variables must be previously computed from the training data, ‘naively’ assuming that the features are mutually independent. From these probabilities, the class of an unclassified pattern is decided taking into account the most likely hypothesis.

#### 4.2.4. Decision Tree

**Figure 7:**
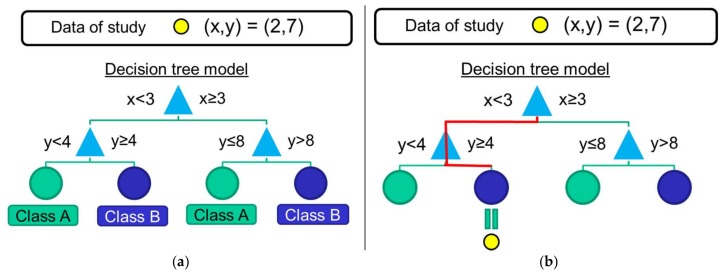
Example of an operation of a decision-tree algorithm: (**a**) after the training phase, the branches and the decision rules the tree are configured; (**b**) during the test phase, the features of an unclassified sample are utilized to apply the decision rules and determine the sample class.

The use of Decision Trees has also been considered by the related literature [50,54,56,57,58]. The basic operation of the algorithm is exemplified in Figure 7, makes use of the training data to create a decision tree that will allow assigning a class to the unclassified mobility patterns. For this purpose, the algorithm selects a certain feature as a decision ‘root’ of the tree while different ‘branches’ are progressively created depending on the values of the other features. The tree is completed when all the features have been considered or when all the training samples in the same branch belong to the same class. The selection criteria of this flowchart-like structure are established to maximize the probability of assigning the correct class to the training data when the different classification rules of the branches are applied.

## 5. Result and Discussion

In this section we analyze the performance of the four afore-described algorithms when they are employed to detect falls in the datasets obtained from the testbed. Apart from comparing the results of the four machine learning techniques, the goal is to explore two elements that impact on the precision of the fall detection process: (1) the selection of the input characteristics for the machine learning techniques, (2) the number and positions of the sensors that are more relevant for the fall identification.

As performance metrics, we utilize the recall or sensitivity (*Se*) and the specificity (*Sp*), which are commonly employed to evaluate the effectiveness of binary classification systems. In contrast with other metrics used in pattern recognition (such as precision or accuracy), *Se* and *Sp* are not affected by the unbalance between the number of existing samples of both types (in this case, falls and ADLs) which are employed to test the detection algorithms.

The sensitivity describes the capacity of the classificatory to correctly identify an event of the ‘positive’ class (here: falls) when this event actually occurs. So, it can be computed as the true positive rate:

(11) $$Se=\frac{TP}{TP+FN}$$

where *TP* indicates the number of ‘True Positives’, i.e., the number of falls correctly labeled by the classifier, while *FN* is the number of ‘False Negatives’ or the number of falls that are misidentified as ADLs. The sum *TP* + *TN* corresponds to the total number of falls in the testing dataset.

Similarly, the specificity informs about the efficacy of the detection system to avoid false alarms (or ‘False Positives’) by properly classifying the actual ADLs. This true negative rate is defined as it follows:

(12) $$Sp=\frac{TN}{TN+FP}$$

where *TN* and *FP* represent the number of ‘True Negatives’ (ADLs which are well identified) and ‘False Positives’ (ADLs mistaken as falls), respectively.

In all the detection schemes, a higher specificity is attained at the cost of decreasing the sensitivity of the system. Therefore, as a trade-off between specificity and sensitivity must be achieved in a FDS, we consider the geometric mean of these two parameters ($\sqrt{Sp\cdot Se}$) as a global metric to characterize the goodness of the detection process.

Once that the performance metric is defined, for the study of these elements we utilize a 2*^k^* factorial design, where *k* designs the number of possible factors (selected input statistic or sensor position) that can affect the detection process.

Every comparison is repeated for the four considered machine-learning based detection techniques. Thus the effectiveness of the algorithms to detect the falls is also compared in a systematic way under a wide range of circumstances. 

To investigate the statistical representativeness of the comparison, the differences in the different results that the alternative use of the factors entails are assessed by means of an ANOVA test. The ANOVA test allows to decide if the means of two or more different populations are equal or not. The test is aimed at determining if different treatments in an experiment cause significant differences in the final results, and consequently, it permits evaluating the importance of the different factors that may alter the operation of the fall detector.

In particular, the factorial ANOVA analyzes the series of residuals resulting from subtracting the values of the obtained observations and the mean of the observations. ANOVA assumes that residuals are independent and follow a normal distribution with a common finite variance (homoscedasticity) [59].

Table 2 details the number of experiments employed for every type of movement (ADLs and falls) as well as the distribution in groups employed for both training and testing with an ANOVA analysis.

To proceed with the supervised learning of the algorithms, the 746 samples obtained from the testbed were divided in a training and a test dataset of 183 and 563 samples, respectively. The number of samples selected to define the training datasets for each type of fall or ADL are described in Table 2. Within the same type, the samples for each phase (training and testing) were randomly chosen.

To apply the ANOVA test, we also divided at random the test dataset into six different ‘blocks’ or ‘subsets’ of approximately 93 samples. We decided to divide the samples into six subsets as long as this number would allow representing the Gaussian nature of the residuals with six points while keeping a population of almost 100 samples in each subset for an adequate estimation of the performance metric.

The number of samples of the same movement type is almost homogeneous for the six sub-sets (as also indicated in Table 2). The values of the specificity (*Sp*) and sensitivity (*Se*) were computed after separately applying the four detection algorithms to the six testing sub-sets for all the possible combinations of the impacting factors (input characteristics and sensor positions). Thus, by replicating the test with the 6 sub-sets, the variance of the series of the performance metric ($\sqrt{Sp\cdot Se}$) measured for the subsets can be investigated.

### 5.1. Analysis of the Impact of the Selection of the Acceleration-Based Features on the Fall Detection Performance

We firstly investigate which characteristic (or set of characteristics) should be selected as input statistics of the machine learning algorithms to maximize the accuracy of the detection decision. For that purpose, we compare the algorithms by analyzing the performance metrics that are obtained when different sets of the input features (described in Section 4.1) are considered. For each set of characteristics, the global specificity and sensitivity are computed by averaging the metrics that are obtained when a specific combination of body sensors are employed. Thus, the specificity ($Sp_{i}$) and sensitivity ($Se_{i}$) for the *i*-th combination of characteristics are calculated as:

(13) $$Sp_{i}=\frac{1}{N_{cs}}\overset{N_{cs}}{\underset{j=1}{\sum }}Sp_{ij}$$

(14) $$Se_{i}=\frac{1}{N_{cs}}\overset{N_{cs}}{\underset{j=1}{\sum }}Se_{ij}$$

where $Sp_{ij}$ and $Se_{ij}$ indicate the specificity and the sensitivity estimated for the *i*-th combination of input characteristics and the *j*-th combination of employed sensors, respectively. In the equations *N_cs_* represents the total number of the possible 31 combinations of the five body sensors, as the algorithms may consider the signals from just one single device to the five sensors ($N_{cs}=2^{5}-1=31$).

To proceed with the ANOVA analysis of the results, we focus the comparison on the series of the geometric means of the specificity and the sensitivity ($\sqrt{Sp_{i}\cdot Se_{i}}$) which are computed for the different sets of test samples (defined in Table 2) and for every combination of input characteristic and considered sensors. The goal is to identify the combination of input features that lead to the best detection performance in a statistically significant way. In case that the best options do not differ significantly, the combination with a lower dimension (i.e., the lower number of features) should be preferred in order to reduce the computational complexity of the system (which is normally intended to be implemented on a wearable device, such as a smartwatch or a smartphone, which can exhibit memory and computation restrictions). This procedure is repeated for the four machine-learning algorithms, whose results are discussed in the following sub-sections.

#### 5.1.1. Results for Support Vector Machine (SVM) Algorithm

We focus on the analysis of the performance of the SVM algorithm when it is alternatively fed with the different possible combinations (2^6^ − 1 = 63) of the six ‘candidate’ input features. 

Firstly, to prove the validity of the ANOVA analysis of the results, we check the assumptions of normality and homogeneity of variance of the residuals (differences between the obtained metrics for every sample sub-test and the global mean value of the metric for each combination of input features).

**Figure 8:**
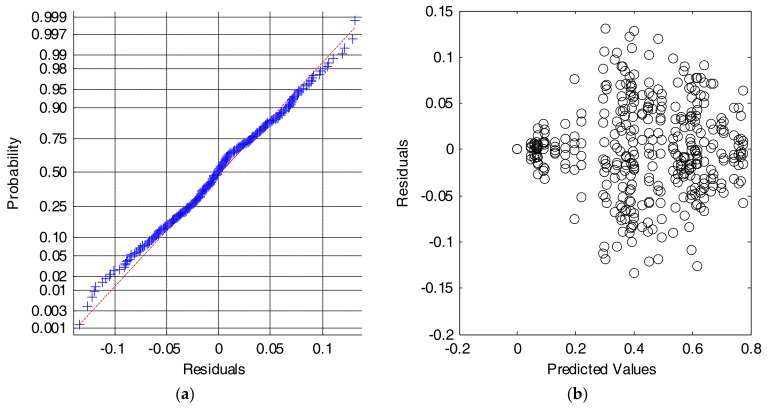
Results for the SVM algorithm. Analysis of the residuals: (**a**) Normal probability plot (crosses: empirical data, dashed line: theoretical normal fit); (**b**) Values of the residuals versus the predicted (or fitted) values.

Figure 8a shows that the Cumulative Distributed Function (CDF) of the residuals can be reasonably approximated by a normal distribution. Similarly, Figure 8b depicts the scatterplot of residuals versus the predicted values (estimated means) of the series of performance metrics obtained for every combination of inputs. The figure illustrates that the variability of the series is only clearly different for those input combinations for which the algorithm presents a very poor behavior. In these cases, where the input features do not feed the algorithm with a convenient characterization of the movements, a very low value of the performance metric (with values of the specificity or the sensitivity close to zero) is achieved, and, consequently residuals also tend to zero. Conversely, this problem does not appear for the rest of combinations, for which the variability of the residuals is homogeneous. This lack of homoscedasticity is not critical for the ANOVA test, provided that the experiments are balanced (all series have the same size) and that the ratio of the maximum to the minimum variances of the series does not exceed a proportion of 4 to 1 [59].

In fact, different data transformations that are recommended by the literature [59,60] when the criterion of a common variance is not completely met were also applied to the data series. As the conclusions drawn with these transformations were basically the same for all the cases, for the sake of clarity, we only show the results obtained with the untransformed series and in the original units.

**Figure 9:**
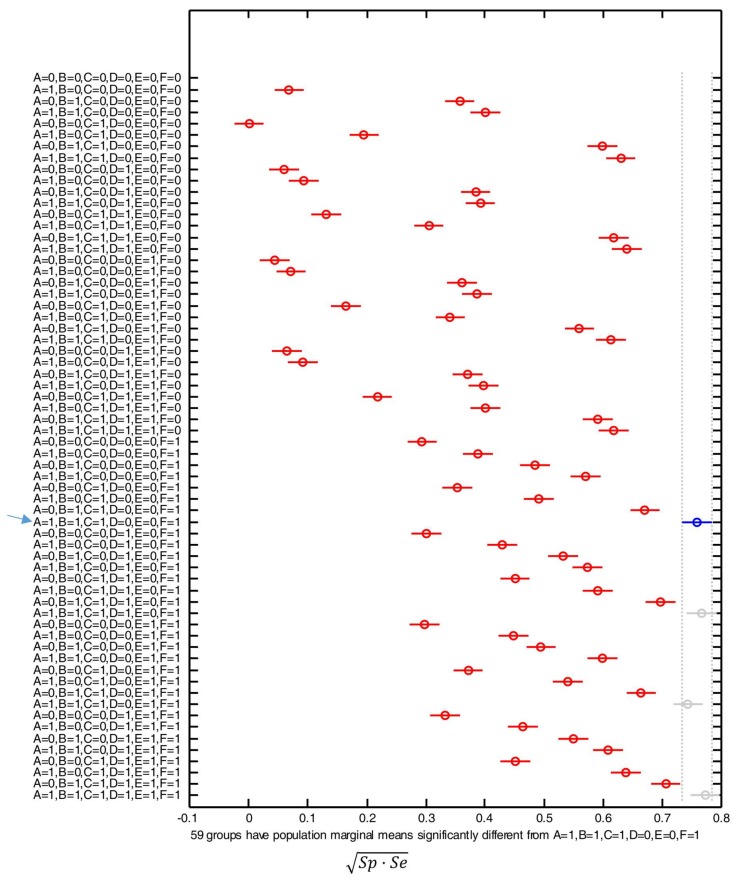
Comparison of the means of the performance metric ($\sqrt{Sp\cdot Se}$ or geometric mean of the sensitivity and the specificity) obtained with the SVM algorithm for all the possible combinations of input features: A = $\mu_{SMV}$, B = $A_{w_{diff\left(max\right)}}$, C = $\sigma_{SMV}$, D = $\mu_{\theta }$, E = $\mu_{SMV_{diff}}$, F = $\mu_{Ap}$. For each combination, the *y*-axis of the figure indicates with ‘1’ or ‘0’ whether the corresponding feature is considered (‘1’) or not (‘0’). The combination with the best results is indicated with a blue arrow.

Figure 9 shows the post hoc multiple comparison of the computed means based on the results generated by the one-way ANOVA analysis. The central marks in the bars of the graph represents the estimated mean while the lines expand to the corresponding comparison interval for a 95% confidence level. 

In the figure the blue mark and line refers to the optimal combination, i.e., that which minimizes the number of required input features while achieving an estimated mean of the performance metric which is not significantly lower than those obtained with the rest of the combinations. The gray color of the marks and lines indicates those combinations that offer a similar performance to the optimal case but requiring a higher number of input features. The red color is in turn utilized to illustrate the results of employing those feature combinations that underperform (when compared to the optimal case) in a statistically significant way.

From the figure, we can observe that the optimal results are generated by the combination that employs $\mu_{SMV}$, $A_{w_{diff\left(max\right)}}$, $\sigma_{SMV}$, and $\mu_{Ap}$ (A = B = C = F = 1, D = E = 0), without considering $\mu_{\theta }$ and $\mu_{SMV_{diff}}$. For the combination of these four inputs, the achieved value of the geometric mean of the specificity and the sensitivity lies in the interval [0.735–0.785], which is significantly different from the result attained with the use of 59 out of the other 62 possible combinations of input features.

According to the ANOVA analysis, Table 3 shows the relative variation (expressed as a percentage) that every single input feature produces in the results, with respect to the global mean of the metric. The Table also includes the same values for the combinations of two inputs ($\mu_{SMV}$ and $A_{w_{diff\left(max\right)}}$, and $A_{w_{diff\left(max\right)}}$ and $\mu_{Ap}$) that also produce the highest variation. As it could be expected, the best global set of inputs is that resulting from combining the four inputs that individually have a higher impact on the result, especially $A_{w_{diff\left(max\right)}}$ (42.312% of variation) $\mu_{Ap}$ (25.807%) and $\sigma_{SMV}$ (14.971%). The interaction of the inputs $A_{w_{diff\left(max\right)}}$ and $\mu_{Ap}$ adds an increase of 3.032%. To characterize the robustness of the results attained by the algorithm, Table 3 also includes the error reported by the ANOVA analysis, which is 5.218%. This error, which should be minimized, may be explained by other factors that have not been considered as input features, such as the small differences in the composition of the test subsets (type and number of executed movements, election and particularities of the experimental subjects for each subset, etc.).

#### 5.1.2. Results for the *k*-Nearest Neighbors (*k*-NN) Algorithm

The previous analysis is repeated for the case in which the detection decision is based on the *k*-Nearest Neighbors (*k*-NN) algorithm. For the application of the algorithm, we set the value of *k* to 9 (nine neighbors). This value, which is selected after different tests, improves the behavior of the algorithm for the employed datasets, as it is close to the number of samples of the different movement types which are employed during the initial training phase of the algorithm.

The results (not depicted here for reasons of space) illustrate again that the CDF of the residuals can be reasonably approximated by a normal distribution while no relevant variation the variability of the residuals is detected for the whole range of predicted values. Consequently, the ANOVA analysis can be considered valid. The post hoc multiple comparison of the performance metric obtained for the different combination of input features indicate that the best performance for the *k*-NN algorithm is achieved when the chosen input characteristics are $\mu_{SMV}$, $\sigma_{SMV}$, $\mu_{\theta }$ and $\mu_{Ap}$. The use of this particular set of characteristics outperforms the results of 59 combinations of the six possible input statistics in a statistically significant way. The particular contribution of the six statistics in the global performance of the detection process is described in Table 4. As it can be observed, the best combination includes the two factors (C = $\sigma_{SMV}$, F = $\mu_{Ap}$) with the highest impact in the results (21.329% and 19.393%, respectively). The high value (6.064%) of the percentage computed for $A_{w_{diff\left(max\right)}}$, which is not included in the best combination of input characteristics, can be actually explained by the negative impact (reduction of the performance metric) that is the achieved if this factor is considered. The table with the conclusions of the ANOVA analysis also informs that the error due to other factors (not considered as inputs) amounts to 21.25%.

#### 5.1.3. Results for the Naïve Bayes Algorithm

As in the previous cases, the visual inspection of the CDF and the variability of the residuals show that the conditions of the normal distribution of the population and the homogeneity of the variances are reasonably met. Again, we assume that the small divergences between the actual CDF and the Gaussian fit can be neglected if we take into account that the model is balanced as the number of samples in each test group is basically the same. Thus, we assume the ANOVA analysis can be reasonably applied as a valid tool. Besides, the post hoc comparison of the method when the different combinations of input features are considered indicate that, for the Naïve Bayes algorithm, the best results are achieved when the systems utilizes as inputs the following characteristics: B = $A_{w_{diff\left(max\right)}}$, C = $\sigma_{SMV}$, D = $\mu_{\theta }$ and F = $\mu_{Ap}$. The performance of the algorithm for this combination of inputs (which yields a global metric in the interval [0.671–0.745]) significantly differs from those obtained with other 48 combinations. Table 5 in turn specifies the individual influence of each possible input characteristics as well as the four combinations of two inputs that presents the highest impact on the results. The table reveals the importance of selecting $\mu_{Ap}$ and $A_{w_{diff\left(max\right)}}$ (and their corresponding combination) on the global performance. On the other hand, the error (due to the presence of other external components that affect the evaluation) computed by the ANOVA analysis is 13.484%.

#### 5.1.4. Results for the Decision Tree Algorithm

The same analysis procedure of the Section 5.1.1, Section 5.1.2 and Section 5.1.3 is applied to the results achieved by the Decision Tree learning method. The normality and the homoscedasticity of the residuals are contrasted and again, the graphs (not depicted here) seem to hold the conditions of the ANOVA test.

From the comparison of the mean performance metrics achieved for the different combinations of input features, we conclude that the best performance of the Decision Tree algorithm takes place when $\mu_{SMV}$, $A_{w_{diff\left(max\right)}}$ $\sigma_{SMV}$, and $\mu_{Ap}$ are used as input variables to characterize the mobility of the individuals. For this combination, the performance metric (the geometric mean of the specificity and sensitivity) is estimated to be in the range [0.801–0.849], which is significantly higher than the intervals computed for most other combinations (53 out of 62).

Table 6 describes the contribution of the different statistics (and the most impacting combinations of two factors) to the global performance of the algorithm. The table highlights the importance of employing, as input parameters, the variance of the standard deviation of the acceleration module ($\sigma_{SMV}$) and, in the second place, $\mu_{Ap}$ to discriminate falls from ADLs.

The error due to non-considered factors is computed to be 12.110%.

#### 5.1.5. Impact of the Election of the Input Characteristics: Summary of the Results

From the study performed in the four previous subsections we can draw the following conclusions:

- The use of a higher number of input characteristics does not necessarily correlate with an enhancement in the behavior of the decision algorithms. For the four strategies under study, no statistically significant improvement (in the geometric mean of the specificity and the sensitivity) is achieved by using more than four inputs. In fact, the performance of some machine learning strategies even deteriorates when the number of considered inputs increases.
- The most frequent statistics in the combinations that yield the best performances of the algorithms are $\sigma_{SMV}$ (which describes the variability of the acceleration module) and $\mu_{Ap}$ (which is linked to the changes in the perpendicularity of the body with respect to the floor plane). The use of $A_{w_{diff\left(max\right)}}$ (which identifies the presence of sudden and brusque changes of the acceleration module) also increases the effectiveness of all the algorithms (except for *k*-NN). Conversely, $\mu_{SMV_{diff}}$ (which characterizes the mean variation of two consecutive samples of the acceleration module during a certain time window) is not included in any of the best combinations of statistics for any algorithm. In any case, the optimal election of the input characteristics clearly depends on the particularities of the employed algorithm. As it can be noted from Table 7, which summarizes the error and the relative impact of the six possible input characteristics on the algorithm’s performance, the importance of the election of each parameter strongly varies from one algorithm to another. A universal set of parameters cannot be proposed to characterize the mobility with independence of the underlying AI technique selected to identify the fall patterns. Thus, results indicate that input characteristics must be carefully designed and individualized for each detection policy.
- SVM obtains the best results, notably outperforming the other algorithms (especially *k*-NN). The next subsection thoroughly investigates this comparison between the machine-learning strategies when other impacting factor (the selected position of the sensors) is considered.

### 5.2. Study of the Importance of the Sensor Position for the Decision of Machine-Learning Fall Detection Algorithms

Taking advantage from the multisensory Body Area Network developed for the testbed, this section investigates the importance of the election of the sensor positions for an adequate detection decision. In particular, we try to assess if the simultaneous use of several wearable sensors may introduce any improvement. This analysis, which is aimed at minimizing the number of sensors that should be transported in a real application scenario, will be carried out for each of the four considered supervised learning algorithms. The input statistics that will be taken into account for each algorithm in order to classify the samples will be those (summarized in Table 7) that were proved to induce a better discrimination between ADLs and falls.

In all the tests, we evaluate the performance of the algorithms (expressed again in terms of the geometric mean of the specificity and the sensitivity) for all the 31 possible combinations of the five sensors of the body area network (from the simplest case where only the signals captured by just one single sensor are considered, to the case where the measurements of the five sensors are utilized as the input for the machine learning technique).

In the comparison, the algorithms are always individually applied for each considered position. Thus, when more than one sensor is employed, an ‘AND’ policy is applied, that is to say, a fall is assumed to have occurred only if it is simultaneously detected by the algorithm in all the considered positions. From some tentative experiments, we checked that the detection based on an ‘OR’ operation (i.e., a fall is presumed if just one sensor detects it, with independence of the results at the other sensing points) is proved to dramatically increase the number of false positives.

The same procedure of the previous section is again utilized to assess the influence of the position and number of sensors. Firstly, it is confirmed that the series of performance metrics that have been obtained meet the assumptions required by the ANOVA analysis. Then, the results of the analysis itself are presented.

#### 5.2.1. Results for the SVM Algorithm

**Figure 10:**
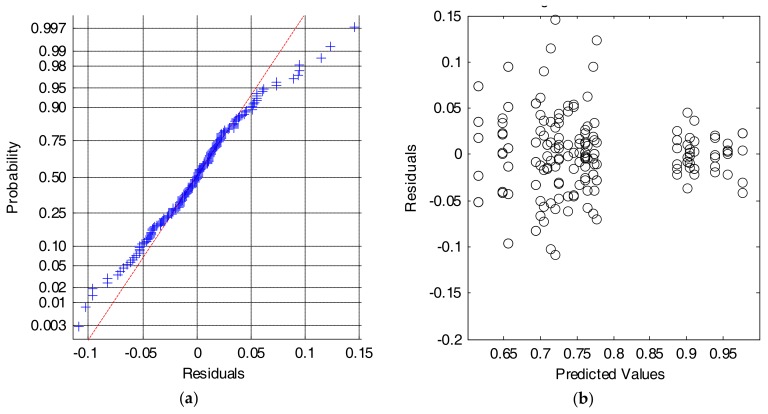
Results for the SVM algorithm. Analysis of the residuals: (**a**) Normal probability plot (crosses: empirical data, dashed line: theoretical normal fit); (**b**) Values of the residuals versus the predicted (or fitted) values.

Figure 10 again illustrates the two graphical tests that can be used to check if the data meet the assumptions of normality and equality of variance. At first glance it is verified that the series do not strictly comply with these conditions. This fact is perceptible in Figure 10b, where the assumption homoscedasticity is shown not to be met completely.

As discussed in the previous sections, there are some possible solutions to reduce this problem. In our case, as recommended in [59,60], we have proved to transform the data (*y*) by applying an exponential function of the type *y^α^*, where α is a constant. This transformation is intended to improve the normality and equality of variance of the numerical series. However, as this experiment is unbalanced, the variations obtained after this transformation do not meaningfully vary. Thus, so we will continue with the ANOVA analysis with the original data to simplify their understanding.

**Figure 11:**
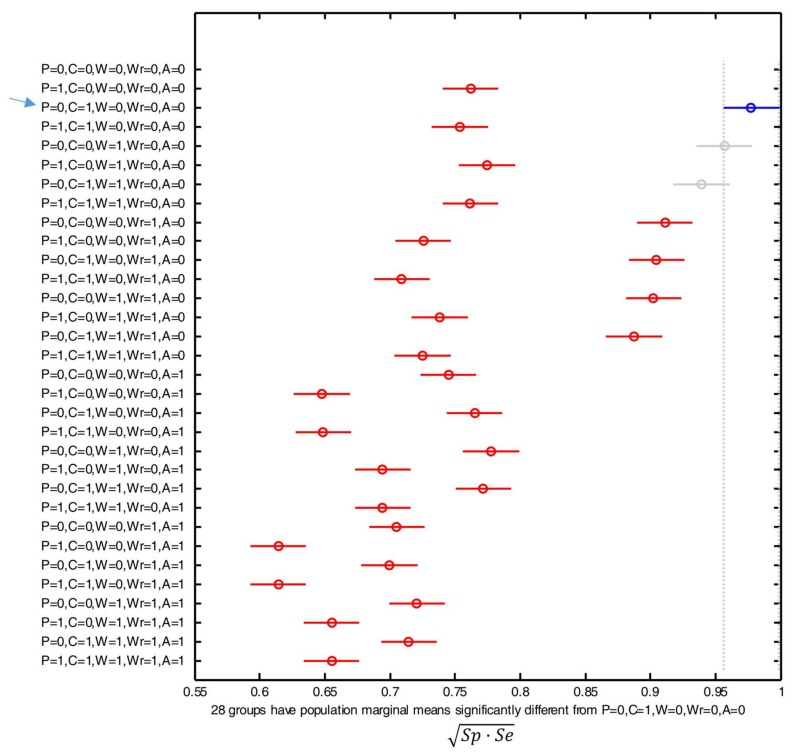
Comparison of the means of the performance metric ($\sqrt{Sp\cdot Se}$ or geometric mean of the sensitivity and the specificity) obtained with the SVM algorithm for all the possible combinations and positions of the sensors. The positions are indicated as: P (Pocket), C (Chest), W (Waist), Wr (Wrist) or A (Ankle). For each combination, the y-axis of the figure indicates with ‘1’ or ‘0’ whether the corresponding sensor is considered (‘1’) or not (‘0’) for the detection decision. The combination with the best results is indicated with a blue arrow.

Figure 11 displays the post hoc multiple comparison of the computed means of the performance metric that resulted from the ANOVA analysis (for which an error of 14.162% was computed), when all the 31 possible combinations of the five BAN sensors are considered. As in the previous section, the figure indicates the estimated mean by depicting a circle in the corresponding bar, which in turn expands to a comparison interval for a 95% confidence level. The blue color is again employed to underline the combination which maximizes the performance, while the red lines correspond to those combinations whose behavior is significantly worse than the optimal case. Gray lines are utilized to identify those combinations whose results present a confidence interval which partially overlap the optimal case (and, consequently, cannot be considered significantly different).

From the results we can infer that the best performance is achieved by using the sensors attached to the waist and chest. Thus, the utilization of any of these two devices as the unique sensor of the system or the combination of both sensors clearly improves the efficiency of the algorithm, as their results significantly differ from the rest. For these three configurations (only-waist, only-chest or the combined use of the sensors on the waist and chest) the computed geometric mean of the sensitivity and the specificity is around 0.95 (which implies false negative and false positive rates lower than 5%). This outperformance could be attributed to the fact that the chest and waist are the sensing points which are closest to the gravity center of the body so that they are less affected by spurious and sudden movements of the limbs. Consequently, the sensors on these positions probably characterize better the mobility of the whole body. Contrariwise, the utilization of the signals captured by the other three devices (the phone in the pocket and the SensorTags on the wrist and on the ankle) do not contribute to the system efficacy and even degrade the algorithm performance. This is particularly true for the case of the sensor on the ankle, as the performance metric plummets below 0.75 for any combination that employs the measurements of the accelerometer located on this point.

#### 5.2.2. Results for the *k*-NN Algorithm

In the case of studying the residuals plots computed when the ANOVA test is applied to the performance metric obtained with the *k*-NN algorithm, we can conclude that the assumptions of normality and homoscedasticity are met. The small variations of the variance as a function of the range of the predicted values can be neglected due to the balanced design of the experiment.

The ANOVA analysis results in an error of 22.857%, which can be justified by the presence of factors different from the position of the sensor, which cause a deviation from the overall average of the experiment, such as the physiognomics and mobility differences of the subjects

From the multiple comparison of the mean values of the performance metric and their corresponding 95% confidence intervals for the different combinations of sensors, we obtain that the best results are again achieved by using the acceleration signals measured at the chest and waist. The differences of the results for these three configurations (only-waist, only-chest and waist-chest) combinations are not statistically significant although they are slightly higher (close to 0.975) than those obtained with the SVM algorithm. Again, the consideration of a more complex BAN (by combining the detection decision on other locations) does not introduce any benefit in the system. Similarly, the use of the signals at the ankle particularly deteriorates the system behavior.

#### 5.2.3. Results for the Naive Bayes Algorithm

The same conclusions as for the previous algorithms can be drawn if we employ a Naïve Bayes strategy to detect the falls. Firstly, the conditions of normality and homoscedasticity of the residuals present a similar behavior to those registered for SVM and *k*-NN algorithms.

The study of the performance metric also reveals that the best option is to consider the detection carried out by the algorithm at the chest, which yields a value of the geometric mean of *Sp* and *Se* in the interval [0.922–0.981]. Similarly, the 95% confidence intervals of the results obtained with the sensor at the waist and with a combination of the sensors on the waist and chest overlaps with that achieved on the chest. Thus, they cannot be considered as poorer. Conversely, the worst performance is again attained whenever the detection process takes into account the application of the algorithm to the signals captured at the ankle. The error of the ANOVA analysis for this algorithm was estimated to be 22.857% (higher than for the precedent algorithms).

#### 5.2.4. Results for the Decision Tree Algorithm

We repeat the testing procedure when the detection algorithm is based on a Decision Tree learning mechanism. We observe again that the criteria of normality and equality of variance of the residuals are not completely met. However, as in the previous experiments, although the data do not strictly comply with these assumptions, the balanced nature of the experiment allows assuming that their influence on the validity of the final results will be limited.

The multiple comparison in turn shows that the optimal operation of the algorithm (with a performance metric in the interval [0.938–0.991]) is also achieved by the use of the signals captured on the chest followed by those of the waist, the combination of waist and chest, the wrist and the combination of wrist and chest. On the other hand, the detection based on the sensors located on the pocket and/or the ankle remarkably deteriorates the effectiveness of the algorithm. Besides, the computed error of the ANOVA analysis was 40.620% (higher than for the precedent algorithms). This is a sign that there are a number of external factors—not considered in the analysis as system variables—that could notably impact on the results.

#### 5.2.5. Summary and Discussion of the Results

In the previous subsections we have assessed the behavior of the four considered machine-learning strategies to detect falls when they are individually applied to the signals produced by the 31 possible combinations of sensors employed in our testbed.

Table 8 summarizes the results obtained by the best combinations that lead to the best results of the geometric mean of the specificity and the sensitivity, as well as the results that are obtained when just a single sensor is employed. The table indicates the sensors that are considered for each combination and highlights in bold those cases in which the performance metric is above 0.9 (which implies that the specificity and sensitivity simultaneously reach values higher than 94.8%). From these results we can draw the following conclusions:

- The particular location of the sensors on the user’s body has a noteworthy influence on the effectiveness of the fall detector with independence of the chosen algorithm.
- The best results are always achieved when the detection algorithm employs the acceleration measurements captured on the chest (or trunk) or waist, the two points that are closest to the gravity center of the human body. This conclusion is coherent with the results of previous works [17,33,61,62,63,64,65,66] that compared the performance of the FDS when operating on two or more positions. In this regard, we cannot forget that ergonomics is a key aspect in the design of any wearable system. The analysis of the state-of-the art on FDSs performed by Thilo et al. in [67] has shown that all the aspects related to ergonomics are almost permanently ignored by the related literature. Thus, for example, these authors prove that the research on FDS prototypes rarely takes into account the opinion of the elderly (the main target of this type of emergency systems). In this vein, chest may constitute a quite unnatural place to locate a FDS as attaching or fixing a sensor to the chest most probably results in some discomfort to the user. Conversely, a belt on the waist (to which the sensors can be easily bonded or stitched) may introduce a more ergonomic alternative.
- For all the algorithms, the worst results are associated to the use of the sensor at the ankle. This can be justified by the fact that the mobility of the ankle does not describe the global stability of the body. Hence, many ADLs can be misidentified as falls (and vice versa) if this location is used as a sensing point.
- The consideration of a trouser pocket as a location for the sensor does not seem to introduce any improvement in the results. The outcome of the detection process is negatively affected if the algorithms take into account the measurements of the accelerometer embedded in a smartphone in the pocket. Pockets may provide users with a comfortable solution to carry small sensors. However, the variability of the typology of the pockets (width, height, ergonomics, internal space, attachment to the body) among the users may impact on the capability of this location to characterize the mobility of the body. Authors in [58] already showed that smartphone-based fall detectors are prone to errors if the device shifts within the pocket. Thus, the use of loose pockets to transport the smartphone may clearly undermine the reliability of this device to implement a FDS. In addition, the motion of the thigh during falls can likely be mistaken by that registered during the execution of certain types of ADLs. This bad behavior of this position forces to reconsider the role of the smartphone as a wearable sensor in fall detection systems. Although many studies have proposed its use in FDSs (taking advantage of its popularity and ease of programming), its effectiveness would be very limited unless it is transported in an uncomfortable (e.g., on a belt) or unnatural place (some works have even tested its effectiveness in FDSs when it is firmly attached to the chest). In the case of the smartphones employed in this study, the sampling frequency of its acceleration sensor was higher than that of the sensor motes (200 Hz vs. 20 Hz), so it can also be deduced that a higher sampling rate does not correlate with a better performance.
- The simultaneous application of the algorithm to the signals sent by more than two sensors never increases the global performance metric (i.e., the possible increase of the specificity never compensates the loss of sensitivity caused by the fact that the fall must be detected in several points). As a consequence, the increase of the complexity of the body sensor network (which undoubtedly affects its ergonomics and cost) is not connected to a higher efficacy of the detection decision and should not be contemplated. The number of sensors is much less important than the election of the sensor position. A single-sensor architecture (with an accelerometer attached to the chest) seems to be enough to maximize the system effectiveness. The use of a second ‘backup’ sensor on the waist does not degrade the performance while it could help to avoid false positives in case of any anomaly suffered by the device on the chest. In this regard, the use of a smartwatch on the wrist as a more natural backup detection point could be also contemplated. In fact, the combined use of a smartwatch and a smartphone has been proved to outperform the effectiveness of the detection decision when compared to a “stand-alone” smartphone-based FDS [68,69,70]. Özdemir has shown in [17] that a fall detection algorithm based on the measurements captured by a sensor on the wrist may lead to a sensitivity higher than 97%.
- If we compare the results achieved by the sensor on the waist (used as a single sensor) for the different algorithms, we can conclude that the best performance is attained by the SVM algorithm (although the confidence intervals obtained for the four machine learning strategies partly overlap). For the case of the SVM, the confidence interval of the performance metric is in the interval [0.956–0.999] with a mean value of 0.9775, which entails minimum mean values for the specificity and the sensitivity higher than 95.5%. In addition, SVM is the algorithm that produces the lowest error (14.162%) for the ANOVA analysis. This lower error reduces the possible impact of other possible factors that have not been considered (e.g., differences in the mobility patterns or physical characteristics of the subject). In any case, the impact of inter subject variability on the results should be studied in future studies. Thus, the ANOVA analysis should be developed taking into account the individual performance of the algorithms when applied to each subject separately. For that purpose, a longer dataset with a higher number of samples per subject is required in order to obtain a better characterization of the performance metrics (sensitivity and specificity) for each participant.

## 6. Conclusions

This article has presented a systematic assessment of the performance of different supervised learning algorithms when they are utilized for the automatic discrimination of falls and ADLs in wearable fall detection system. The study is based on the analysis of a dataset (UMAFall) obtained through a testbed with experimental subjects. In order to generate the mobility data traces, the subjects were requested to executed a series of predefined movements (ADLs and mimicked falls) while wearing a multisensory wireless Body Area Network, consisting of a Bluetooth network with five inertial mobility units (embedded in four commercial sensing motes and a smartphone) located on five different parts of the body. In our framework, the quality metric selected to weigh up the efficiency of the algorithms to detect falls was the geometric mean of the specificity and variance, which can be considered a good trade-off between the needs of avoiding false negatives (falls identified as ADLs) and false positives (ADLs mistaken as falls).

The purpose of the analysis was twofold. Firstly, we intended to assess the impact of the election of the statistics employed (as input variables for the algorithms) to describe the evolution of the subjects’ mobility during the movements. Secondly, we evaluated the importance of the location of the sensors for the detection effectiveness of the machine learning strategies.

The final results enable quantifying the contribution of the different factors involved in the detection of a fall and discerning if they lead to significant changes in the final performance of the system.

As it refers to the characterization of the accelerometer signals, it has been shown that each detection mechanism needs a different combination of input characteristics to achieve its best performance. Nevertheless, there are statistics that augment the effectiveness of all the algorithms whenever they are considered: the standard deviation of the acceleration module (measured during a time window around the acceleration peak), which describes the variability of the human mobility, and the mean module of the non-vertical acceleration components, which characterizes the alterations of the perpendicularity of the subjects’ body with respect to the floor plane.

In any case, results suggest that a greater number of input features does not necessarily lead to a better operation of the machine learning algorithms. In fact, in many cases, this increases in the complexity of the system and even produces a decrease in the measured performance metric.

Besides, it has been evidenced that the location of the employed sensors strongly influences the efficacy of the FDS. In this regard, for all the studied algorithms, the best results correspond to the detection based on the signals captured on the chest and/or waist (the two considered sensing points which are closest to the center of mass and gravity of the human body). In addition, it has also been inferred that the combined use of several sensors (aimed at decreasing the number of false alarms) does not improve the performance, since it heavily decreases the sensitivity. The study has also shown that a sensor in a trouser pocket (the position that is conventionally utilized by for many users to transport a smartphone) is not the best option for an adequate discrimination rate. Consequently, a fall detection system merely based on a smartphone (as it is massively proposed by many papers in the literature) is not feasible as long as the phone should be constantly fixed to a belt or ‘worn’ in a quite unnatural way (firmly attached to the chest).

Another important contribution of the paper is the employed methodology as the validity of all the comparisons was evaluated through an ANOVA analysis. Thus, it has been possible to determine the statistical significance of the differences obtained when the algorithms were tested under different configurations of the detection process (input variables, selection of the sensors). The study of the statistical significance of the results (an aspect that is normally neglected by the existing bibliography about FDSs) is completed by the information reported by the error computed by the ANOVA analysis. This error can be used as a metric to evaluate the robustness of the algorithms with respect to the presence of other impacting factors that have not been taken into consideration for the analysis (such as the personal characteristics of the subjects).

Many mid- and low-end smartphones are still manufactured without gyroscopes. Thus, as the smartphone is a key element in our architecture, this study has only focused on the discrimination of falls based on signals captured by the accelerometers. However, the trunk angle velocity and trunk orientation have been proved to be two key parameters to detect anomalies in the mobility of the body during the moments immediately preceding the impact [71]. Our future research plans to extend this study by incorporating metrics derived from the signals measured by the gyroscope embedded in the sensors. So, the contribution of the information on the changes of the body orientation to the fall detection process could also be evaluated.

The analysis of the performance of the fall detection schemes of this work was developed in an offline mode (by processing the samples on a computer using Matlab scripts), as the general goal was to assess the ‘abstract’ effectiveness of the machine learning strategies under different circumstances. Therefore, the problems related to the implementation of this type of architectures in a wearable system (with inherent hardware and software limitations) were outside the scope of our study. However, these aspects (which are normally ignored by the literature) should also be systematically investigated.

Future studies should analyze in detail the implementability of systems that combine wireless communications with external sensors and complex artificial intelligence techniques that must make decisions in real time.

Battery-powered wearable units acting as the core of a Body Area Network for a FDS may have serious shortcomings to provide the computational resources (computing speed, memory, etc.) required by the communication dynamics and the algorithm of the fall detection service. In the case of using a conventional smartphone as the center of the sensor network, the practical coexistence of the detection application and the other typical activities executed by the device (making calls, messaging, browsing, etc.) should be also studied in detail. Otherwise, the use of a smartphone as a specific tool exclusively intended for fall detections (as it is conceived in those architectures where the phone is firmly attached to the user’s chest) is pointless. In this context, battery drain due to FDS applications should be also studied in detail.

## Figures and Tables

**Table 1:** Personal characteristics of the participants in the testbed.

Subject ID	Gender	Age	Height (cm)	Weight (kg)
Subject 1	Female	67	156	76
Subject 2	Female	22	167	63
Subject 3	Male	68	168	97
Subject 4	Male	27	173	90
Subject 5	Male	24	179	68
Subject 6	Male	24	175	79
Subject 7	Male	28	195	81
Subject 8	Female	22	167	57
Subject 9	Male	55	170	83
Subject 10	Male	19	178	68
Subject 11	Male	26	176	73
Subject 12	Female	51	155	55
Subject 13	Female	18	159	50
Subject 14	Female	22	164	52
Subject 15	Male	26	179	67
Subject 16	Male	21	173	77
Subject 17	Female	27	166	66
Subject 18	Male	24	177	66
Subject 19	Female	23	163	93
Mean value		31.26	170.53	71.63
Standard deviation		15.98	9.54	13.52
Median value		24	170	68

**Table 2:** Typology and number of the movements executed by the experimental subjects during the testbed. The table also indicates the number of samples utilized for both training and testing phases during the validation of the machine learning algorithms.

Movement Type	No. of Executions	No. of Training Samples	No. of Test Samples (for the 6 Sub-Sets)					
Applauding	42	14	4	4	5	5	5	5
Raising both arms	43	14	5	5	4	5	5	5
Emulating a phone call	46	14	5	5	5	5	6	6
Opening a door	43	14	5	5	5	5	4	5
Sitting on a chair and getting up	64	14	9	9	8	8	8	8
Walking	63	14	8	9	8	8	8	8
Bending	59	14	7	7	8	8	8	7
Hopping	53	14	6	6	6	7	7	7
Lying down on/standing up from a bed	57	14	7	7	7	7	7	8
Going upstairs and downstairs	40	7	6	5	5	5	6	6
Jogging	28	9	3	3	3	3	4	3
Forwards fall	71	13	10	10	10	10	9	9
Backwards fall	73	14	9	10	10	10	10	10
Lateral fall	64	14	8	8	9	9	8	8
**Total**	**746**	**183**	**92**	**93**	**93**	**95**	**95**	**95**

**Table 3:** Error reported by the ANOVA analysis and relative influence of the election of the input features on the global result when the Support Vector Machine (SVM) algorithm is applied. Inputs: A = $\mu_{SMV}$, B = $A_{w_{diff\left(max\right)}}$, C = $\sigma_{SMV}$, D = $\mu_{Ap}{}_{\theta }$, E = $\mu_{SMV_{diff}}$, F = $\mu_{Ap}$.

Feature	%
A	3.332
B	42.312
C	14.971
D	0.701
E	0.207
F	25.807
A&B	0.785
B&F	3.032
Error	5.218

**Table 4:** Error reported by the ANOVA analysis and relative influence of the election of the input features on the global result when the *k*-Nearest Neighbor (*k*-NN) algorithm is applied. Inputs: A = $\mu_{SMV}$, B = $A_{w_{diff\left(max\right)}}$, C = $\sigma_{SMV}$, D = $\mu_{\theta }$, E = $\mu_{SMV_{diff}}$, F = $\mu_{Ap}$.

Feature	%
A	0.954
B	6.065
C	21.329
D	4.120
E	1.334
F	19.393
Error	21.250

**Table 5:** Error reported by the ANOVA analysis and relative influence of the election of the input features on the global result when the Naïve Bayes algorithm is applied. Inputs: A = $\mu_{SMV}$, B = $A_{w_{diff\left(max\right)}}$, C = $\sigma_{SMV}$, D = $\mu_{\theta }$, E = $\mu_{SMV_{diff}}$, F = $\mu_{Ap}$.

Feature	%
A	0.977
B	11.552
C	7.472
D	12.604
E	2.143
F	32.782
A&F	1.668
B&D	1.494
B&F	4.067
C&F	1.361
Error	13.484

**Table 6:** Error reported by the ANOVA analysis and relative influence of the election of the input features on the global result when the Decision Tree algorithm is applied. Inputs: A = $\mu_{SMV}$, B = $A_{w_{diff\left(max\right)}}$, C = $\sigma_{SMV}$, D = $\mu_{\theta }$, E = $\mu_{SMV_{diff}}$, F = $\mu_{Ap}$.

Feature	%
A	3.709
B	6.775
C	41.181
D	1.450
E	1.158
F	14.913
C&F	2.178
D&E	1.979
Error	12.110

**Table 7:** Error reported by the ANOVA analysis and relative influence of the election of the six considered input features on the global result for the four learning machine strategies. The symbol (✓) or (-) indicates if the corresponding feature is included (or not) in the combination that achieves the (statistically significant) best performance.

	$\mu_{SMV}$		$A_{w_{diff\left(max\right)}}$		$\sigma_{SMV}$		$\mu_{\theta }$		$\mu_{SMV_{diff}}$		$\mu_{Ap}$		Error
**SVM**	**✓**	**3.332%**	**✓**	**42.312%**	**✓**	**14.971%**	-	0.701%	-	0.207%	**✓**	**25.807%**	5.218%
***k*-NN**	**✓**	**0.954%**	-	6.065%	**✓**	**21.329%**	**✓**	**4.120%**	-	1.334%	**✓**	**19.393%**	21.250%
**Naive Bayes**	-	0.977%	**✓**	**11.552%**	**✓**	**7.472%**	**✓**	**12.604%**	-	2.143%	**✓**	**32.782%**	13.484%
**Decision Tree**	**✓**	**3.709%**	**✓**	**6.775%**	**✓**	**41.181%**	-	1.450%	-	1.158%	**✓**	**14.913%**	12.110%

**Table 8:** Summary of the results of the study on the impact of the sensor position. For every combination, the tick symbol (**✓**) identifies those sensors that are employed by the algorithm.

	Trouser Pocket	Chest	Waist	Wrist	Ankle	$\sqrt{Se\cdot Sp}$
**SVM** Error = 14.162%.	**✓**	-	-	-	-	[0.741–0.783]
	-	**✓**	-	-	-	**[0.956–0.999]**
	-	-	**✓**	-	-	**[0.935–0.978]**
	-	-	-	**✓**	-	[0.890–0.933]
	-	-	-	-	**✓**	[0.724–0.766]
	-	**✓**	**✓**	-	-	**[0.918–0.961]**
***k*-NN** Error = 22.857%	**✓**	-	-	-	-	[0.833–0.875]
	-	**✓**	-	-	-	**[0.950–0.993]**
	-	-	**✓**	-	-	**[0.950–0.992]**
	-	-	-	**✓**	-	**[0.903–0.946]**
	-	-	-	-	**✓**	[0.797–0.840]
	-	**✓**	**✓**	-	-	**[0.926–0.969]**
**Naive Bayes** Error = 16.967%	**✓**	-	-	-	-	[0.792–0.853]
	-	**✓**	-	-	-	**[0.922–0.981]**
	-	-	**✓**	-	-	**[0.903–0.963]**
	-	-	-	**✓**	-	[0.863–0.899]
	-	-	-	-	**✓**	[0.614–0.675]
	-	**✓**	**✓**	-	-	[0.863–0.923]
**Decision Tree** Error = 40.620%	**✓**	-	-	-	-	[0.787–0.840]
	-	**✓**	-	-	-	**[0.938–0.991]**
	-	-	**✓**	-	-	**[0.913–0.965]**
	-	-	-	**✓**	-	[0.890–0.943]
	-	-	-	-	**✓**	[0.825–0.878]
	-	**✓**	**✓**	-	-	[0.896–0.949]
